# Nebraska State Dental Society

**Published:** 1878-01

**Authors:** J. F. Roseman

**Affiliations:** Recent Secretary


### NEBRASKA STATE DENTAL SOCIETY.

The Annual Meeting of the Nebraska State Dental Society was held at Lincoln, Tuesday, July 24th, 1877, and continued two days. The following essays were read and discussed: Dental Hygiene, by Dr. Sanborn, "Our Wards" Dr. Roseman, Sympathetic and replex influences between the teeth and other organs by Dr. King. The election of officers for the ensuing year resulted as follows:

| | | |
|---|---|---|
| President, | Dr. | S. H. King, Lincoln; |
| Vice " " | " | D. A. Vance, Nebraska; |
| Corresponding Secretary, | " | J. W. Chadduck Neb. City; |
| Secretary and Treasurer, | " | W. F. Roseman, Fremont; |

| Executive Committee, | Commitee on membership. |
|---|---|
| Dr. A Billings, | Dr. J. W. Chadduck, |
| " T. C. Kern, | " D. A. Vance, |
| " W. F. Roseman, | " J. F. Sanborn, |

Adjourned to meet at Fremont the fourth Tuesday, in July, 1878.

J. F. Roseman, Recent Secretary.

